# Crosstalk Communications Between Islets Cells and Insulin Target Tissue: The Hidden Face of Iceberg

**DOI:** 10.3389/fendo.2022.836344

**Published:** 2022-02-03

**Authors:** Allan Langlois, Aurore Dumond, Julie Vion, Michel Pinget, Karim Bouzakri

**Affiliations:** Centre européen d’étude du Diabète, Unité Mixte de Recherche de l’Université de Strasbourg « Diabète et Thérapeutique », Strasbourg, France

**Keywords:** Beta cell (B-cell), insulin secretion, islets, skeletal muscle, adipose tissue, Liver

## Abstract

The regulation of insulin secretion is under control of a complex inter-organ/cells crosstalk involving various metabolites and/or physical connections. In this review, we try to illustrate with current knowledge how β-cells communicate with other cell types and organs in physiological and pathological contexts. Moreover, this review will provide a better understanding of the microenvironment and of the context in which β-cells exist and how this can influence their survival and function. Recent studies showed that β-cell insulin secretion is regulated also by a direct and indirect inter-organ/inter-cellular communication involving various factors, illustrating the idea of “the hidden face of the iceberg”. Moreover, any disruption on the physiological communication between β-cells and other cells or organs can participate on diabetes onset. Therefore, for new anti-diabetic treatments’ development, it is necessary to consider the entire network of cells and organs involved in the regulation of β-cellular function and no longer just β-cell or pancreatic islet alone. In this context, we discuss here the intra-islet communication, the β-cell/skeletal muscle, β-cell/adipose tissue and β-cell/liver cross talk.

## Introduction

Maintaining glucose homeostasis requires pancreatic islets cells’ secretion of several hormones including insulin by β-cells, glucagon by α-cells, somatostatin by δ-cells and the pancreatic polypeptide (PP) by PP-cells. Glycaemia regulation is also allowed thanks to several insulin sensitive/responsive tissues like liver, adipose tissue and skeletal muscle (1, 2). Specifically, β-cells releasing insulin, a hypoglycaemic hormone, play a critical role in this physiological regulation. Indeed, defective insulin secretion is the cause of all forms of diabetes (3).

Diabetes in its two main forms is characterized by an absolute or relative insulin deficit. Several signals are thought to lead to impaired cell function and possibly a decrease in cell mass in type 2 diabetes (T2D), with autoimmune cell destruction underlying type 1 diabetes (T1D). More precisely, T1D results from the destruction of pancreatic β-cells that is mediated by the immune system. Multiple genetic and environmental factors found in variable combinations in individual patients are involved in the development of T1D. It was recently described that the two first auto-antibodies which initiate the autoimmune process are insulin autoantibodies or glutamic acid decarboxylase autoantibodies. Furthermore, the autoimmune response is affected by environmental factors such as nutrition etc… (4). T2D concerns the majority of diabetic patients (around 90–95%) and is due to the development of insulin resistance that can result in a progressive loss of β-cell insulin secretion (5). 

Insulin resistance is defined as the inability of cells to respond normally to the insulin, leading to a decrease in glucose uptake in primary insulin-sensitive organs such as skeletal muscle and adipose tissue. Moreover, hepatic insulin resistance impairs suppression of glucose production by insulin in hepatocytes, participating in the chronic increase in glycaemia characteristic of diabetes (6, 7). In parallel with alterations in glucose metabolism, insulin resistance induces an accumulation of circulating free fatty acids due to an increase in adipose tissue lipolysis and hepatic *de novo* lipogenesis (8). This contributes to ectopic fat deposition in liver or skeletal muscle, which in turn exacerbate insulin resistance. Usually, it appears that insulin resistance onset involves multiple and complex pathways, with inter-linking and multidirectional effects (9). These mechanisms include inflammation, lipotoxicity, endothelial reticulum stress and mitochondrial dysfunction (10). Moreover, insulin resistance plays a larger role in T1D pathological process than it is generally recognized. Along with the increasing incidence of T1D, obesity and physical inactivity have steadily increased in children and adolescents. The role of insulin resistance in T1D has only recently been accepted.

Interestingly, the insulin resistance alone does not induce T2D. Indeed, healthy β-cells are able to compensate this insulin resistance by increasing in number and enhancing their secretory capacities (11). Thus, insulin resistance, in addition to impaired β-cell function, is a hallmark of T2D. Therefore, for a long time, β-cell was considered as the main target for the treatment of diabetes. However, it is now well established that optimal glycaemic regulation involves cross communication between several cell type, organs and tissues such as intra-islet cells, intestine L-cells, pancreas, liver, skeletal muscle, adipose tissue etc… (12–14). Moreover, for adaptation to nutritional and environmental conditions, this close conversation is essential. Therefore, to treat diabetes mellitus, it seems important to consider not only a cell type but all the organs involved in this glycaemic regulation. In addition, it is widely described that this inter-organ communication involves many metabolites, including myokines, adipokines, hepatokines, isletokines, extracellular vesicles (EV) etc… (12–14). To prove the importance of this communication network, numerous studies have shown that any disruption of it induces metabolic dysfunctions and metabolic syndromes such as obesity and diabetes (12–15).

In this review, we sought to illustrate with current knowledge how β-cells communicate with other cell types and organs in physiological and pathological contexts. Moreover, this review will provide a better understanding of the microenvironment and the context in which β-cells exist and how this can influence their survival and function. In this context, we discuss here the intra-islet communication, the β-cell/skeletal muscle, β-cell/adipose tissue and β-cell/liver cross talk.

## B-Cell Communication in Pancreatic Islet

In response to glucose elevation, β-cells secrete insulin through the canonical insulin pathway involving GLUT2 transporter, K_ATP_ channels, intra-cellular Ca ^2+^ increasing etc…. Conversely, at low levels, β-cells employ a negative feedback mechanism to help maintain the blood glucose within a safe range (16–18). However, it is clearly established that β-cell insulin secretion mechanism is even more complex and is regulated also by a close inter-organ/cells cross-talk involving various metabolites and/or physical connections. This communication is also found into the islet.

### β-Cell to β-Cell Communication

For decades, the challenge has been to determine how β-cells finely regulate their insulin secretion linked to glucose concentration and whether this resides in a collective response *via* a close communication between β-cells.

#### Role of “Hub Cells” in β-Cell Function

The arrangement of β-cells within pancreatic islets plays a critical role for insulin secretion through the generation of rhythmic activity. Indeed, Johnston NR et al. demonstrated that the islet functional architecture is composed of hub β-cells with pacemaker properties. Moreover, authors showed that silencing of hubs abolished coordinated islet responses to glucose, whereas specific stimulation restored communication patterns (19). Activation of these “Hub cells”, recently renamed “Leader cells” (20), may trigger [Ca ^2+^]_i_ waves that diffuse to the other cells within the islets, called “Followers cells”. Then, the coordinated increases and decreases in [Ca ^2+^]_i_ drive pulses of insulin secretion (21, 22). Interestingly, it was demonstrated that these “Hub cells” are targeted by pro-inflammatory and glucolipotoxic factors inducing β-cells dysfunction (19). Therefore, these “Hub cells” are essential for regulating synchronization of islet insulin secretion and any disruption of it could contribute to diabetes onset. Nevertheless, Satin LS et al. have recently reported that depolarization of “Hub cells” is sufficient to trigger electrical activity and [Ca ^2+^]_i_ waves but it is difficult for one cell to supply enough current to repolarize the entire islet. Thus, authors suggest the involvement of diffusible factors released by specialized cells or groups of cells within islets like nitric oxide, carbon monoxide, GABA … but more studies are necessary to conclude (22).

#### Role of Gap Junction in β-Cell Function

It is widely described in the literature that Gap junctions provide one of the micro-anatomical bases for appropriate glucose-induced insulin release (23, 24). Indeed, β-cell is known to express gap junctions involved in cell coupling and in the exchange of ions and small metabolites between β-cells (24–26). Particularly, Speier S et al. highlighted an important role for Cx36-gap junctions in modulating stimulation threshold and kinetics of insulin release (24). Moreover, it was shown that coupling of β-cells improves insulin synthesis and secretion, while uncoupling leads to altered β-cell function (27). Very recently, in order to improve knowledge about the exact role and effects of β-cell/β-cell communication on glucose homeostasis, Boris P et al. developed a new study model. This consists on a dynamical network model in which N network nodes represent individual β-cells and network links represent couplings with k neighboring cells (18). Thanks to their study model, they demonstrated the existence of a glucose-induced transition in β-cell activity thanks to increasing coordination through gap-junctional signaling and paracrine interactions. In conclusion, insulin β-cell response to glucose stimulation is a collective and coordinated action involving Gap junctions.

#### Role of EVs in β-Cell Function

Among elements involved in β-cell to β-cell communication, it is well established that EVs play a critical role and can influence β-cell function (28). Indeed, Javeed N et al. have recently showed that pro-inflammatory β-cell EVs induce a complete loss of insulin secretion in response to glucose and promote a pro-inflammatory islet transcriptome (29). More precisely, Guay C et al. demonstrated that these effects are mediated by miRNA derived-Evs, which are transferred to neighboring β-cells. Indeed, down-regulation of the miRNA-mediating silencing protein Ago2 in recipient cells prevent deleterious effect of non-coding RNAs (30).

In conclusion, EVs transfer is an important cell-to-cell communication mechanism regulating β-cell function and constitute targets of interest to develop therapeutic strategies.

### Crosstalk Between β-Cells and α-Cells

α-cells, with β-cells, are among the two most abundant and essential endocrine cell type in the pancreatic islet for the maintenance of glycaemia balance. The role of α-cells is to release glucagon into the bloodstream in order to increase blood glucose levels in opposition to β-cells that secrete a hypoglycaemic hormone (31). Thus, they were considered for long time as functional antagonists but the function of these two cellular types is influenced by each other. Indeed, glucagon produced by the pancreatic α-cell stimulates β-cell function while insulin has an inhibitory effect on glucagon release (32). This cross communication between β and α-cells enables active regulation to maintain stable blood glucose concentration (15, 31, 33). For that, β-cells release some inhibitory factors of α-cellular function including insulin, Zn^2+^, ATP and γ-aminobutyric acid (31, 34). Conversely, α-cells produce, in addition of glucagon, factors which affect the regulation of β-cells activity. These can have both autocrine and paracrine signaling properties (15, 31).

Rodriguez-Diaz R et al. have recently shown that glucagon input increases insulin secretion also from the neighboring β-cell in human pancreatic islet (35). For that, authors transplanted human islets into the anterior chamber of the eye of diabetic nude mice. Once restoring normoglycaemia, they inhibited human glucagon receptors with a specific antagonist (L-168,049). Then, they showed that this treatment decreased insulin secretion from human islet grafts and increased glycaemia to pre-diabetic levels. Consequently, for the first time, it was demonstrated that insulin secretion has to be amplified by input from adjacent α-cells (35).

Furthermore, in intestinal L-cells, proglucagon, release by α-cells, is converted to GLP-1 by PC1/3 that is able to potentiate insulin secretion under conditions of elevated blood glucose concentration (36). Moreover, some studies suggest that GLP-1 may be also directly produced in the α-cell, also through PC1/3 expression, in order to increase insulin secretion (37–39). Recently, it was shown that this α- to β-cell communication and the subsequent enhancement of insulin secretion are lost in KO mice for proglucagon peptides in the α-cell. The same observation was done if the receptors for proglucagon peptides (GLP-1R and GCGR) are silenced (37, 40, 41). Therefore, β- to α-cell communication seems currently attributed to proglucagon products but further studies are necessary to clarify the exact contribution of glucagon and GLP-1 in this communication and the mechanisms involved.

Finally, Human α-cells secrete acetylcholine, which strongly potentiates the glucose-induced insulin secretion (42, 43). Indeed, human α-cells provide paracrine cholinergic input to surrounding endocrine cells, whose β-cells. Particularly, human α-cells express acetylcholine transporter, release acetylcholine in response to glucose concentration and amplify β-cell answer to increase glucose concentration (43).

In conclusion, a very close and complex relationship exists between the two cell types that is fundamental for setting the regulation of insulin secretion and thus for glucose homeostasis, involving the secretion of various metabolites. Moreover, α/β-cell communication is not only a direct but is also an indirect crosstalk involving other intermediate cell types as intestine. Consequently, any cell dysfunction can alter this communication between α- and β-cells and thus can affect insulin secretion in response to glucose elevation. Therefore, for the development of therapeutic strategies it is necessary to be not only interested in the communication between two cell types but also in the impact that other organs/cells could have on this communication. This increases sharply the task.

### Communication Between β- and δ-Cell

Other component of pancreatic islet cyto-architecture are the δ-cells. With α- et β-cells, these 3 cell types constitute the endocrine functional part of the islet which are finely connected to allow an adequate response to glycaemic variations (44–46). δ-cell is an important paracrine regulator of β and α-cell’s secretory activity by secreting somatostatin. More precisely, somatostatin is an inhibitor of both glucagon and insulin release and so, is an important regulator of glucose homeostasis (46). It acts through different isoforms of the somatostatin receptor (47, 48). Furthermore, the interaction between δ-cells and other intra-islet cells also becomes defective in diabetes. Indeed, this defect reduces paracrine feedback to β-cells to exacerbate hyperglycemia or enhanced inhibition of α-cells, disabling counter-regulation, to cause hypoglycemia (49).

δ-cell release somatostatin in response to high glucose and to local-acting signaling molecules secreted by islet cells, such as acetylcholine, glutamate, urocortin3 (Ucn3), ghrelin (48, 50–53). In addition, β- and δ-cells can regulate glucagon secretion by α-cells through gap junction communication (54).

Interestingly, Arrojo e Drigo R et al. have shown that δ-cell structure is composed of filopodia, which play a crucial role in β-cell function regulation. Indeed, authors observed that these filopodia are dynamic structures that contain a secretory machinery, enabling the δ-cell to reach a large number of β-cells within the islet (46). Furthermore, they showed that endogenous IGF-1/VEGF-A signaling modulates this. Therefore, any disruption in this regulation may contribute to early stages of beta cell failure and diabetes pathophysiology (46, 55).

In order to bring new knowledge on intra-islet cells communication, a recent study from Germany aimed to determine how α- and β-cells regulate somatostatin secretion (56). For that, authors studied in transgenic mice models the effects of varying glucose concentrations together with infusions of arginine, glucagon, insulin and somatostatin, as well as infusions of antagonists of insulin, somatostatin and GLP-1 receptors. Interestingly, they demonstrated that somatostatin and glucagon secretion are linked in a reciprocal feedback cycle with somatostatin inhibiting glucagon secretion at low and high glucose levels. Moreover, they observed that glucagon stimulates somatostatin secretion thanks to glucagon and GLP-1 receptors activation. Conversely, they showed that insulin or activation of its receptor did not affect somatostatin secretion. Therefore, glucagon pathway plays a crucial role on somatostatin secretion and could be an interesting therapeutic target to control somatostatin secretion as this hormone is a strong regulator of glucagon and insulin secretion.

### Relationship Between β-Cell Function and Intra-Islet Endothelial Cell

Pancreatic islets are highly vascularized and composed of fenestrated capillaries. This dense and tortuous vascular network is essential to deliver quickly insulin in the bloodstream in response to glucose elevation. Indeed, islets receive 6–20% of the pancreas’s direct arterial blood flow, although they only represent 1–2% of the organ mass (57–59). Moreover, the connection between endothelial cells and endocrine cells enables proper gas and nutrition’s exchange and waste removal *via* the bloodstream.

The existence of cross-talk between β-cells and intra-islet endothelial cells which play a crucial role in β-cell development and function providing non-nutritional signals to islets, has been well established for decades (58, 60). For example, few years ago it was shown that endothelial cell signals regulate the expression of transcription factors in order to initiate dorsal pancreas development by selectively inducing the transcription factor Ptf1α (61). Moreover, using transgenic mice, Lammert E et al. discovered that vessels not only provide metabolic sustenance, but also provide inductive signals inducing insulin expression and islet hyperplasia (62). Among these signals, the main ones is the Vascular Endothelial Growth Factor A (VEGF-A) known to promote endothelial migration and proliferation after binding to its receptor (63). Thus, a defect in VEGF-A signaling can impairs β cell proliferation, insulin secretion and glucose homeostasis (58, 64). Furthermore, Hepatocyte Growth Factor (HGF) produced by intra-islets vessels, also regulates β-cell proliferation. Moreover, it is essential for β cell differentiation, function, and proliferation (58). Then, it was demonstrated that markers of endothelial cell function (E-selectin, Il6, endothelin-1 and endothelial nitric oxide synthase) are overexpressed in islet endothelial cells from diabetic db/db mice and the exposure of these molecules on pancreatic islets decreases insulin secretion during a glucose stimulation test (GSIS) and decreases insulin content. Thus, authors highlighted that in diabetes, islet endothelial cells have a dysfunctional phenotype, which may contribute to loss of β-cell function (65, 66). Finally, it is widely described that endothelial cells modulate β-cell function in mature islets by secreting basement membrane components that interact with specific receptors in β-cells (67–70). For example, Daniel B et al, showed the existence of an effective paracrine interaction between islets microcapillary endothelial cells and β-cells that modulates glucose-induced insulin secretion *via* the TPI-sulfonylurea receptor-KATP channel (SUR1-Kir6.2) complex attenuating interactions (70).

Interestingly, β-cells also release factors, which influence intra-islet endothelial cell function. Indeed, Figliolini et al. demonstrated that biologically active islet-derived EVs are able to shuttle anti-apoptotic and pro-angiogenic mRNAs and miRNAs into endothelial cells, which is a promising target to improve islet transplantation (71).

On this part, the role of intra-islet endothelial cells is not only to deliver quickly hormones into the bloodstream to regulate glucose homeostasis but is also to influence the function and survival of cells through a close communication with β-cells involving secreted molecules. In conclusion, this is a two-way communication and any endothelial and/or β-cell dysfunction will participate in diabetes onset.

### Cellular Plasticity in the Pancreas

Cellular plasticity in the pancreas also plays a crucial role in glycaemia homeostasis and it represents a strong illustration of pancreatic cell communication. Interestingly, maintaining normoglycaemia involves a certain degree of pancreatic islet’s biological adaptation. For example, an increased demand for insulin can lead to an increase in β-cell mass. Moreover, any changes in these adaptive mechanisms can lead to the incapacity in maintaining normoglycaemia and to the development of diabetes (72).

It is well described that pancreatic cells are able to transdifferentiate in functional β-cells to compensate insulin secretion impairment to maintain normoglycaemia. Indeed, it was demonstrated that pancreatic α-cells and δ-cells become insulin expressers upon ablation of insulin-secreting β-cells, promoting diabetes recovery (73). Interestingly, in young mice, δ-cells (but not α-cells) undergo spontaneous conversion into β-cells, proceeding through a dedifferentiated intermediate (74, 75). Moreover, it was recently shown that α-cells and PPY-producing γ–cells, obtained from deceased non-diabetic or diabetic human donors, can be lineage-traced and reprogrammed by the transcription factors Pdx1 and MafA to produce and secrete insulin in response to glucose (73, 76). Furthermore, Furuyama K et al. demonstrated that transplantation of human α-cells converted in β-cells in diabetic mice reverses diabetes and that the graft remains functional for 6 months (73). However, in diabetic patients it was observed a β-cell plasticity and a loss of β-cell identity. Thus, it is proposed that β-cells fail to maintain a fully differentiated glucose-responsive and drug-responsive state in diabetic individuals with poorly controlled and long-lasting periods of hyperglycemia (77). Then, it has been demonstrated that β-cell neogenesis occurs *via* transdifferentiation of acinar or ductal cells, differentiation of progenitors to β-cells in exocrine and endocrine tissue or by replication of preexisting β-cells (78, 79).

Finally, this concept of cellular plasticity is primordial to understand the importance of the role of pancreatic cell intercommunication in β-cell function and glycaemia regulation. Indeed, the loss of cell identity due to transdifferentiation process will disturb intra and inter pancreatic cell communication (described all along this chapter) essential for optimal β-cell function. Thus, it represents another way of interest for future diabetic patients’ treatments.

## Crosstalk Between B-Cell And Skeletal Muscle

Skeletal muscle is the largest insulin-sensitive organ in the body, so it plays a major role in postprandial glucose homeostasis. Consequently, altered insulin action in skeletal muscle can lead to a pathological state of insulin resistance, in which normal concentrations of insulin induce an impaired biological response (80). Interestingly, Dr Bente Klarlund Pedersen was the first to suggest that skeletal muscle is as an endocrine organ. Indeed, she suggested that certain cytokines and other peptides, called “myokines”, were produced, expressed and released by muscle fibers (81). Moreover, several studies have shown that the contraction of skeletal muscles releases a selected panel of myokines, which can act hormonally both locally and in distant tissues. For example, these secreted myokines exert specific endocrine effects on visceral or median fat and have direct anti-inflammatory effects (82–84). Other myokines act locally within muscle *via* paracrine mechanisms, exerting their effects on signaling pathways involved in lipid metabolism (83).

Skeletal muscle tissue is composed of a heterogeneous population of muscle fibers ranging from slow contraction type I fibers to fast contraction type IIx/d fibers (85). Type I fibers will use lipids for their function, while type IIx/d fibers will mostly use glucose (86, 87). Recently, our laboratory showed that skeletal muscle cells can secrete different myokine profiles depending on their insulin sensitivity and fibrillar composition and can impact β-cell function and survival (12).

### Impact of Human Skeletal Muscle Cell Secretome on Pancreatic β-Cells

Our team showed that skeletal muscle with different insulin sensitivity can have a differential impact on pancreatic β cell function and survival (12). Moreover, our work demonstrated that myokines secreted by insulin-sensitive skeletal muscle increased pancreatic β-cell proliferation as well as glucose-induced insulin secretion. In contrast, myokines secreted by insulin-resistant skeletal muscle induce loss of insulin secretion and destruction of pancreatic β-cells. In conclusion, skeletal muscle secretome contains factors that can have either a positive (insulin-sensitive muscle) or a negative (insulin-resistant muscle) impact on pancreatic β-cells. For this review, we have chosen to discuss about cytokines and chemokines independently tested for their effects on pancreatic β cells.

#### Cytokines and Chemokines

For example, IL-1β alone can have a positive or a negative impact on β-cell function, survival and proliferation. Furthermore, these differential effects are depending on its concentration and on its duration of exhibition (88). In addition, we have also shown positive effects on β-cell survival and proliferation for low levels of IL-1β secreted by the cells themselves when cultured on a particular extracellular matrix (89, 90).

Moreover, we showed that TNF-α, at high concentration, decreases insulin secretion induced by glucose and stimulates apoptosis. However, as observed with IL-1β, at low concentration, TNF-α promotes β-cell proliferation and improves insulin secretion. Thus, this indicates the existence of bimodal effects for this cytokine (12).

Then, the interferon-gamma-inducible protein (IP-10), also called C-X-C motif chemokine ligand 10 (CXCL10), is increased in the secretome of insulin-resistant human skeletal muscle cells (12). In addition, its circulating level is increased in the serum of patients with type 1 and type 2 diabetes (91, 92). This has led several groups to study the impact of this chemokine on the endocrine pancreas. The expression of CXCL10 in the pancreas was first shown to accelerate the autoimmune process (93). Secondly, a direct impact of CXCL10 on the function and survival of pancreatic β cells was shown with induction of their apoptosis, altered insulin secretion and decrease in insulin mRNA.

Studies on the effects of IL-6 on β-cell function have shown mixed results, with some finding a negative effect (94) and others a positive effect on insulin production (95, 96). Furthermore, it appears that these effects are more complex than a direct effect. Indeed, IL-6 plays an essential role in a muscle-entero-pancreatic communication loop. During exercise IL-6 releases GLP-1 from L cells in the intestine and further improves β-cell function through local production of GLP-1 in α-cells, leading to better glycaemic control (97). Interestingly, high and acute secretion of IL-6, observed during exercise, have beneficial effects. However, chronic elevation of plasma IL-6 is associated with negative clinical parameters, including the development of type 2 diabetes (98).

#### Follistatin

A decade ago, it was shown that plasma follistatin is rapidly elevated during physical activity, peaks during the recovery phase and remains elevated for a few hours (99). The origin of exercise-induced follistatin appears to depend on the type of exercise performed. During resistance exercise such as weight training, follistatin mRNA expression increases in skeletal muscle tissue biopsies from women on hormone replacement therapy (100). The same observation was made in healthy young men after a session of strength training (101). However, recently we have shown that follistatin is also secreted by the liver in response to exercise. This follistatin secreted into the bloodstream can then target the pancreas and regulate the secretion of insulin and glucagon. The latter will subsequently target the liver and in turn regulate the secretion of follistatin (102). This example perfectly illustrates the complexity of the role played by the inter-organs communication on β-cell function and survival.

#### Fractalkine

Another myokine has generated great interest in the treatment of diabetic patients, fractalkine, also known as CX3CL1. It is a CX3C chemokine expressed in various cell types such as skeletal muscle cells and pancreatic β-cells (103–105). Furthermore, knockdown of CX3CR1 (fractalkine receptor) in a mouse model was found to induce hyperglycaemia and to reduce nutrient-stimulated insulin secretion. In addition, the injection of fractalkine into C57BL/6N mice made it possible to potentiate their β-cell function, to increase plasma insulin levels and to improve their glucose tolerance (106). In another study conducted by our team, we showed that fractalkine protects human β-cells from the harmful effects of TNFα on the molecular mechanisms involved in the trafficking of insulin granules. We then highlighted that this myokine restored the phosphorylation and expression of key proteins involved in the insulin secretion pathway such as AKT, AS160, paxillin, IRS2 for example (105). In summary, all these data suggest that the Fractalkine-CX3CR1 axis could be a target of interest for treating diabetes and that fractalkine could be an interesting pharmacological candidate for treating diabetic patients.

#### Osteoprotegerin

Recently, we have highlighted a set of myokines secreted by glycolytic skeletal muscles. These myokines, such as osteoprotegerin (OPG), a member of the TNF receptor superfamily, are being evaluated therapeutically for replacement of pancreatic β-cells destroyed in T1D. Indeed, recent studies have shown that OPG enables the replication of human pancreatic beta cells by modulating the CREB and GSK3 pathways, by binding RANKL and thus, interfering with the RANKL/RANK antiproliferative interaction. Furthermore, it has been shown by plasma insulin assay and a glucose tolerance test that the glycemic balance is significantly improved in diabetic mice treated with 1.0 mg/g of mOPG-Fc (107). In addition, we recently demonstrated in a study carried out on primary cultures of human myotubes, that OPG is a specific myokine of the triceps and that it significantly decreases the apoptosis of pancreatic β-cells. Finally, we found that OPG counteracts both the negative effects of cytomix and TNFα on primary pancreatic β-cell proliferation and insulin secretion (108).

In conclusion, far from being an inert tissue in terms of inter-organ communication, skeletal muscle secretes myokines, which can affect the function of distant organs/tissues either favorably or unfavorably. Here, we have summarized the potential impact of myokines in the communication between skeletal muscle and endocrine pancreas. This is a new route of communication that we believe is further altered by the degree of insulin resistance in skeletal muscle. Finally, the identification of the skeletal muscle secretome may have important implications for understanding the decrease in the functional mass of β cells in diabetes and for developing an innovative therapy.

## Crosstalk Between B-Cell and Adipose Tissue

For decades, white adipose tissue has long been considered a mere storage tissue. In 1990s, it has been highlighted that adipose tissue is an endocrine tissue able to secrete heterogeneous bioactive factors including proteins (i.e. adipokines), lipids (i.e. lipokines) and extracellular vesicles (e.g. exosomes). Since the 1990s, the number of adipose tissue’s secretions has continuously increased and new factors are regularly identified. All of these secretions establish communications with a variety of organs and cells including the pancreas and particularly β-cells. A lot of original papers and reviews have already been published concerning the impact of the “classic” factors (e.g. leptin, adiponectin) (13). For these reasons, this review focuses on recently described adipose tissue’s secretions that crosstalk with β-cells.

### Adipokines

#### Asprosin

Asprosin, identified as a novel adipokine in 2016, is a C-terminal product generated by the cleavage of a proprotein by activated protease furin, which generates mature fibrillin-1 and 140 amino-acid asprosin (109). Plasma asprosin level increases during starvation to stimulate hepatic gluconeogenesis and prevent hypoglycaemia. An increase is also observed in patients with obesity and T2D (110, 111). Interestingly, asprosin is negatively correlated with homeostasis model assessment for β-cells function (HOMA-β) (112) that indicates that asprosin may be involved in β-cells dysfunction during T1D. Indeed, it has been recently shown that treatment of MIN6 cells by asprosin increases caspase 3 activity as a marker of apoptosis and decreases cell viability (113). This is associated with an impairment of GSIS. Surprisingly, it has been shown that asprosin secretion is enhanced by irisin, an exercise-induced myokine (114) which raises the question of its beneficial or deleterious effect. So, it will be necessary to elucidate more precisely asprosin’s mechanisms. Then, this observation highlights the idea of a complex and indirect crosstalk between organs that can influence β-cells function. All of these results suggest asprosin as a potential therapeutic target for preserving pancreatic β-cells.

#### Adipsin

Furthermore, Adipsin or complement factor D was the first described adipokine and plays a central role in metabolism (115). This adipokine is also synthesized by the liver but adipose tissue remains the major source of its secretion. Adipsin stimulates the production of C3a, a component complement able to enhance insulin secretion by increasing the concentration of cytosolic Ca^2+^ in β-cells (116). Interestingly, adipsin expression in adipose tissue is decreased in T2D patients with β-cell failure compared to T2D patients (116). Recently, Gomez-Banoy et al. found that treatment of transplanted pancreatic islets with an adeno-associated virus (AAV) expressing adipsin preserves β-cell mass by inhibiting death in a model of T2D mice (117). Moreover, adipsin maintains β-cell transcription identity and so inhibits dedifferentiation observed during diabetes. Despite these attractive results, to date no study has investigated the role of adipsin in the context of T1D. This adipokine could be an attractive pharmacological candidate and this path deserves to be explored in the future.

#### Secreted Frizzled-Related Protein 5

Finally, Secreted frizzled-related protein 5 (Sfrp5) is an endogenous inhibitor of Wnt signaling pathway identified as a novel anti-inflammatory adipokine in 2010 (118). Typically, its adipose tissue mRNA expression is reduced in rodent models of obesity (118). Moreover, KO mice fed with a high fat diet develop systemic metabolic dysfunction, such as glucose intolerance or adipose tissue inflammation. In 2019, Carstensen-Kirberg et al. showed that treatment of INS-1E cells with Sfrp5 during 24h dose dependently increased GSIS (119). This is associated with a decrease in the phosphorylation levels of c-jun N terminal kinase (JNK), a deleterious pathway for β-cell function (120). In light of these first observations, Sfrp5 appeared to represent a potential target for diabetes mellitus and it seems necessary to deal with it in depth.

### Lipokines

In addition to adipokines, adipose tissue is also able to secrete bioactive lipids called lipokines. By their nature, there factors are difficult to isolate and still few of them have been characterized. Among them, some have shown interesting impact on β-cells and more will certainly be identified in the years to come.

#### Palmitoleate

Palmitoleate was the first lipid identified as being released from adipose tissue and having metabolic effects on distant organs. It is mostly derived from *de novo* lipogenesis and acts as an insulin sensitizer in liver and skeletal muscle tissues (121). Concerning its impact in pancreatic β-cells, it seems to stimulate insulin secretion for high glucose levels (122) but mechanisms of action have not yet been elucidated. Now, further studies are needed to understand the link between palmitoleate and diabetes and to consider this factor as a biomarker or a possible treatment.

#### Fatty Acid Esters of Hydroxy Fatty Acids

Then, Fatty acid esters of hydroxy fatty acids (FAHFA) are a novel class of lipids, which showed beneficial metabolic and anti-inflammatory effects (123). The most well characterized FAHFA species are palmitic acid esters of hydroxyl stearic acid (PAHSA) whose serum and adipose tissue levels are decreased in insulin-resistant subjects (124). In 2019, Syed I et al. showed beneficial impacts of PAHSA on β-cells. Indeed, chronic treatment of NOD mice delays the onset and reduces the incidence of diabetes (125), associated with an enhancement of GSIS. In accordance with that, PAHSA increases proliferation and attenuates cytokine-induced MIN6 cells death. Interestingly, these effects are mediated in part by GLP-1R, widely described as activator of insulin secretion (126). Taken together, these results highlight a new class of potential candidates for treatment of diabetes.

Besides white adipose tissue, brown adipose tissue (BAT) is also able to secrete proteins called batokines. By its ability to increase energy expenditure, targeting BAT to treat diabetes is a particularly attractive strategy. However, the secretome of this tissue remains poorly characterized and nowadays there is no evidence of a direct dialogue between BAT and pancreas during diabetes. The identification and characterization of batokines could open new areas of research and potentially future treatment options.

### Extracellular Vesicles

EV are lipid bilayer particles naturally secreted by cells into the extracellular space. They envelop and release intracellular molecules including proteins, microRNA and bioactive lipids to mediate cell-to-cell communication. Several studies have shown that EV proteins and microRNAs content were altered during diabetes (127, 128). In this way, Gesmundo I et al. showed that EV from human lean adipose tissue promote survival and insulin secretion of β-cells while EV from human obese adipose tissue alters these same settings (129). The authors have not investigated the cargo of the EV but it could be interesting to identify modulated factors in this context. Future studies of adipose tissue-pancreas crosstalk will take into account this important mode of communication.

## Crosstalk Between B-Cell And Liver

The liver is composed of many different cell types: parenchymal cells representing 80% of the liver, including hepatocytes and bile duct cells, and non-parenchymal cells including Kupffer cells (liver macrophages), hepatic stellate cells and sinusoidal endothelial cells (14). For a long period, the secretory role of liver was mainly known for its regulation of coagulation and hemostasis, however the different cells composing the liver give a wide range of secreted proteins, thus its endocrine role in metabolic diseases has started to be described recently (14). Furthermore, its structure composed of sinusoids enable to deliver the liver secretome to the peripheral organs through the central veins and the inferior vena cava (14), making liver one of the largest metabolic organ (130). Indeed, the liver has also an impact on β-cells, principally by regulating the compensatory effect on islets induced in insulin-resistant state. Here, we focus on different liver secretory products that have an impact on β-cells and which represent a real therapeutic interest.

### Hepatokines

#### FGF21

FGF21 was first identified in 2000 principally in the liver (131) but was first described as a metabolic regulator in 2005 (132), involved in lipid metabolism, by decreasing adipose tissue lipolysis, increasing fatty acid oxidation and reducing hepatic lipids. Secondly, it is involved in glucose metabolism by decreasing plasma glucose, which mainly explain its higher circulating expression in patients with obesity and/or hepatic steatosis as a compensatory effect (14, 133). In addition to its metabolic regulator role, FGF21 has been reported as a potential anti-diabetic therapy thanks to its ability to improve β-cell function and mass (132). This anti-diabetic role is explained by different mechanisms: decrease in plasma glucose and triglyceride levels which reduces β-cells’ glucolipotoxicity and activate AKT signaling pathway which improves insulin sensitivity (14, 134, 135).

#### SerpinB1

SerpinB1 is a protease inhibitor mainly expressed in hepatocytes (136). SerpinB1 inhibits particularly the neutrophil elastase that interferes with β-cell proliferation by inhibiting the phosphorylation of proteins involved in insulin or IGF-1 signaling pathways, such as MAPK3 or GSK. Thus, SerpinB1 increases β-cells proliferation particularly in insulin-resistant state (137).

#### Hepatocyte Growth Factor

HGF is expressed in liver in response to ERK signaling and induce its signal through its tyrosine-kinase receptor Met (138). The treatment of β-cells by HGF increases the phosphorylation of IRS2, AKT and ERK (138). This increase is the response of MET activation by HGF, which induce the formation of a MET-Insulin receptor complex responsible of an increase in insulin signal (136, 138, 139).

#### Selenoprotein P

Selenoprotein P is a secretory protein predominantly expressed by the liver (14, 140), which plays an important role in selenium metabolism (141). Selenoprotein P expression induces insulin resistance and impairs glucose metabolism in hepatocytes through the inhibition of AMPK activity (140, 141). Selenoprotein P high level is also involved in the reduction of β-cells and α-cell mass and in the re-arrangement of the position of β-cells and α-cells in the pancreas that might explain the pancreatic insulin level decrease observed when selenoprotein P is overexpressed (141).

### miRNA

miRNAs are small RNA sequences (around 20 nucleotides) that regulate gene expression by binding on mRNAs to suppress translation or induce their lysosomal degradation (14, 142). Thus, intracellular accumulation of miRNAs could specifically regulate metabolic functions such as insulin secretion of β-cells (14, 143).

For example, miR-7218-5p is expressed in hepatocellular EV and its expression is decreased in high fat diet rats EV (130). miR-7218-5p regulates β-cells’ (MIN6) proliferation as a compensatory effect in an insulin resistance state (130). This impact on β-cells’ proliferation is induced by miR-7218-5p’s regulation of Cd74 gene expression. Cd74 is a transmembrane protein involved in immunological processes but also in cell proliferation regulating ERK1/2 and AKT signaling pathway (130).

Finally, other miRNA secreted by the liver are known to impair insulin secretion in β-cells as miR-375, miR-9, miR-143 (14).

### Liver-Brain-Pancreas Network System

Previously, it has been shown that the liver exerts effects on β-cells through its secretory proteins or extracellular vesicles. However, liver can also have an impact on β-cells indirectly. Indeed, ERK pathway is over-expressed in liver of obese mice and increases β-cell proliferation (136). Imai J. et al. have highlighted that ERK over-expression in hepatocytes regulates β-cell proliferation through splanchnic nerves that deliver a signal to the brain which transfers this signal to the β-cells through vagal nerves (136, 144). The vagal nerves release different neural factors, as acetylcholine, adenylate cyclase activating polypeptide (PACAP), vasoactive intestinal polypeptide (VIP), that are going to activate β-cell proliferation through FoxM1 pathway upregulation, which is a critical factor for β-cell mass expansion (136).

Thus, liver also have indirect effect on β-cells through this liver-β-cells inter organ neuronal network involved in β-cell proliferation in insulin-resistant and obese state (136).

### Hepatic/α-Cells Axis

On another hand, liver is also involved in the regulation of glucagon, through a hepatic/α-cells axis (145). α-cells produce glucagon, which, by binding onto the hepatic glucagon receptor, induces hepatic glucose’s production through gluconeogenesis. However, in case of interrupted glucose signalling, a decrease of amino acid (AA) catabolism in the liver is observed increasing amino acid in the blood circulation (145, 146). AA’s increase is involved in α-cell proliferation particularly through glutamine, which is transported in α-cells through Slc38a5 (AA transporter) and, which increases mTOR expression involved in α-cell proliferation (145, 146). This glutamine-dependent α-cell proliferation re-establishes the production of glucagon and, so hepatic glucose’s production (147). Thus, this axis presents several target for diabetes’ therapies (146).

## Crosstalk Between B-Cell and Gut Microbiota

The gastrointestinal tract houses a complex population of microorganisms called gut microbiota. In physiological conditions, it offers many benefits to the host including nutrient and drug metabolism, immunomodulation or maintenance of the structure of gut integrity (148). Dysbiosis of the gut microbiome has been implicated in various diseases including diabetes. Recently, gene sequencing of fecal samples from healthy and diabetic people showed an increase in pro-inflammatory bacteria and a decrease in anti-inflammatory bacteria with diabetes (149). It is also interesting to point out a decline in bacteria that produce short chain fatty acid (SCFA), some of which have shown beneficial effects on the pancreas. Indeed, Pingitore et al. showed that long-term colonic propionate delivery improves β-cell function by potentiating insulin secretion in response to glucose and by protecting cells from cytokine- and palmitate-induced apoptosis (150). In accordance with that, it has been shown that acetate, another SCFA, is able to diminish the frequency of autoimmune T cells in the pancreas and so protect against T1D (151). Finally, intestinal lysozyme can also release Nod1 ligands from commensal bacteria and promote insulin granule transport in β-cell thanks to the recruitment of the protein Rab1a (152). In conclusion, all of these data highlight the importance of taking into account the gut microbiota in the regulation of β-cell function.

## Conclusion

**Figure 1:**
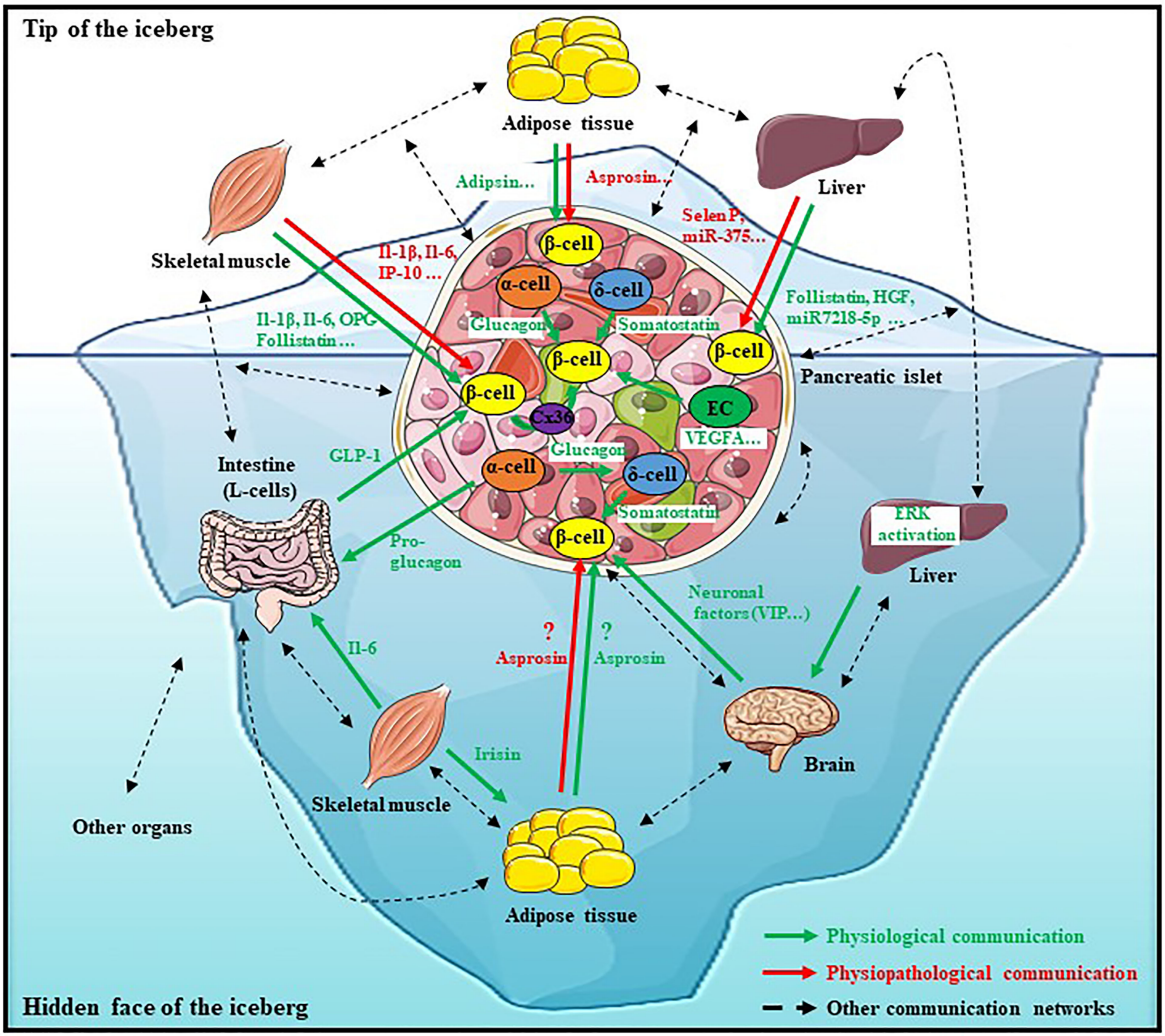
The iceberg of β-cell’s communication network. The physiological β-cellular function is finely regulated by a dense and complex communication network including direct and indirect interactions with various cell types and organs. This communication takes place thanks to various factors secreted from many organs such as the liver, adipose tissue, skeletal muscle, intestine or even the brain which constitute a strong union to allow a physiological β-cell function. However, this description is not exhaustive, this network is more complex and should also involve other organs (Dotted black arrows). Any disturbance in this communication network can lead to dysfunction of β-cell function and to metabolic diseases such as diabetes onset. Therefore, for new anti-diabetic treatments’ development, it is necessary to consider the entire network of cells and organs involved in the regulation of β-cellular function and no longer just the tip of the iceberg.

In this review, we sought to provide recent knowledge on how β-cell function is regulated. First of all, it is important to highlight that this regulation is more complex as some had thought. Indeed, it is largely demonstrated that β-cell insulin secretion is regulated also by a direct and indirect inter-organ/inter-cellular communication involving various factors, illustrating the idea of “the hidden face of the iceberg” (**Figure 1**). Then, this very close and complex relationship between all these cell types and organs is fundamental for setting the regulation of insulin secretion and thus for glucose homeostasis. Moreover, this review provides a better understanding of the microenvironment and the context in which β-cells exist. All of these recent studies showed how this communication can influence β-cell survival, function and that any disruption on this physiological communication can participate on diabetes onset. Therefore, for the development of new treatments for diabetic patients, it is necessary to consider the entire network of cells and organs involved in the regulation of β-cellular function and no longer than β-cell or pancreatic islet alone. Finally, many metabolites and signaling pathways, which improve insulin secretion, could be targeted to treat diabetes but further investigations are required to propose the best therapy.

## Author Contributions

AL, AD, and JV wrote the review. MP and KB edit the text. KB designed and edited all the review. All authors contributed to the article and approved the submitted version.

## Conflict of Interest

The authors declare that the research was conducted in the absence of any commercial or financial relationships that could be construed as a potential conflict of interest.

## Publisher’s Note

All claims expressed in this article are solely those of the authors and do not necessarily represent those of their affiliated organizations, or those of the publisher, the editors and the reviewers. Any product that may be evaluated in this article, or claim that may be made by its manufacturer, is not guaranteed or endorsed by the publisher.

## Glossary

**Table:**

PP	Pancreatic Polypeptide
T2D	Type 2 Diabetes
T1D	Type 1 Diabetes
GLUT2	Glucose transporter 2
Cx36	Connexin 36
ATP	Adenosine TriphosPhate
GLP-1	Glucagon Like Peptide-1
PC1/3	Proprotein convertase 1/3
GLP-1R	Glucagon Like Peptide-1 Receptor
GCGR	Glucagon Receptor
Ucn3	Urocortin3
IGF-1	Insulin-like Growth Factor-1
VEGF-A	Vascular Endothelial Growth Factor-A
Ptf1α	Pancreas transcription factor 1 subunit alpha
Il-6	Interleukin-6
GSIS	Glucose-Stimulated Insulin Secretion
TPI	Triosephosphate Isomerase
SUR1	SulfonylUrea receptor 1
Il-1β	Interleukin-1β
TNFα	Tumor Necrosis Factor α
IP-10	interferon-gamma-inducible protein 10
CXCL10	C-X-C motif chemokine ligand 10
CX3CL1	chemokine [C-X3-C motif] ligand 1
CX3CR1	CX3C chemokine receptor 1
AS160	AKT substrate of 160 kDa
IRS2	Insulin receptor substrate 2
OPG	Osteoprotegerin
CREB	C-AMP Response Element-binding protein
GSK3	Glycogen Synthase Kinase-3
RANKL	Receptor Activator of Nuclear Factor Kappa-B Ligand
RANK	receptor activator of nuclear factor kappa-B
HOMA-β	Homeostasis model assessment-β
C3a	complement component 3a
AAV	Adeno-Associated Virus
Sfrp5	Secreted frizzled-related protein5
JNK	c-Jun N-terminal kinase
FAHFA	Fatty Acid esters of Hydroxy Fatty Acids
PAHSA	Palmitic Acid esters of Hydroxyl Stearic Acid
EV	Extracellular Vesicles
BAT	Brown Adipose Tissue
FGF21	Fibroblast Growth Factor 21
MAPK3	Mitogen-activated protein kinase 3
GSK	Glycogen Synthase Kinase
HGF	Hepatocyte Growth Factor
ERK	Extracellular signal-Regulated Kinase
AMPK	5’ Adenosine Monophosphate-Activated Protein Kinase
EC	Endothelial Cell
Selen P	Selenoprotein P
PACAP	adenylate cyclase activating polypeptide
VIP	Vasoactive Intestinal Polypeptide
